# Rituximab is associated with accelerated dialysis independence in anti-glomerular basement membrane disease: a retrospective cohort analysis of renal survival

**DOI:** 10.3389/fimmu.2026.1835889

**Published:** 2026-05-11

**Authors:** Yulin Wang, Zihan Zhai, Liuwei Wang, Qiuhong Li, Yanhong Guo, Lu Yu, Rong Gou, Lin Tang

**Affiliations:** 1 Department of Nephrology, Zhengzhou University First Affiliated Hospital, Zhengzhou, Henan, China; 2 Zhengzhou University, Zhengzhou, Henan, China

**Keywords:** anti-GBM disease, anti–glomerular basement membrane disease, glomerulonephritis, renal survival, rituximab

## Abstract

**Objective:**

This study aimed to evaluate the efficacy of rituximab (RTX) in the treatment of anti-glomerular basement membrane disease (anti-GBM disease) and to analyze the clinicopathological factors associated with long-term renal outcomes.

**Methods:**

This study enrolled a total of 66 patients with confirmed anti-GBM disease. Patients were divided into two groups: the control group (n=52) received conventional therapy, including plasma exchange, glucocorticoids, and cyclophosphamide as clinically indicated, and the RTX group (n=14) received RTX in addition to the same conventional therapy. The baseline characteristics, treatment responses, and long-term outcomes were compared between the two groups.

**Results:**

The baseline clinical and pathological characteristics were balanced and comparable between the two groups. Although the RTX group demonstrated only numerical advantages in the absolute rate of dialysis independence (50.0% vs. 21.05%, p=0.107) and the 3-year ESRD incidence (42.86% vs. 63.46%, p=0.223), the median time to dialysis independence was significantly shorter in the RTX group compared to the control group (26 vs. 41 days, p=0.009). Kaplan-Meier survival analysis indicated a trend towards higher renal survival in the RTX group, but the difference was not statistically significant (HR = 0.640, p=0.169). Prognostic factor analysis indicated that worse baseline renal function, anemia, hypoalbuminemia, elevated BNP, initial dialysis dependency, and absence of cyclophosphamide treatment were associated with progression to ESRD at 3 years.

**Conclusion:**

In patients with anti-GBM disease, the addition of RTX to standard therapy was associated with accelerated recovery of renal function, as evidenced by a shorter time to dialysis discontinuation. These findings suggest a potential renal recovery benefit of RTX, warranting further validation in larger, prospective studies.

## 1 Introduction

Anti-glomerular basement membrane disease (anti-GBM disease) is a rare yet highly aggressive autoimmune disorder caused by the production of autoantibodies targeting the glomerular basement membrane (1). Its epidemiological profile is distinct, with an annual incidence of approximately 1-1.5 cases per million population, categorizing it as a genuine rare disease (2). Anti-GBM disease is a medical emergency that requires prompt recognition and treatment. Typical clinical features include rapidly progressive glomerulonephritis (e.g., hematuria, proteinuria, and rapidly deteriorating renal function) and pulmonary hemorrhage, which may lead to life-threatening dyspnea and hemoptysis (2). Diagnosis relies on the detection of high-titer anti-GBM antibodies in serum, rapidly progressive glomerulonephritis, and pulmonary hemorrhage (3). When anti-GBM disease is strongly suspected, treatment should be initiated immediately, even before confirmatory test results are available (3). The current standard regimen consists of triple immunosuppressive therapy with the primary goal of rapidly removing pathogenic antibodies from the circulation and suppressing their further production (4).

Rituximab(RTX) is a chimeric human-murine monoclonal antibody that induces selective depletion of mature B cells in peripheral blood and tissues through mechanisms including antibody-dependent cell-mediated cytotoxicity (ADCC), complement-dependent cytotoxicity (CDC), and direct induction of B-cell apoptosis (5). Given that anti-GBM disease is an autoimmune disorder driven by autoantibodies specifically targeting GBM antigens, it is theorized that RTX, by eliminating B cells, may suppress the production of pathogenic antibodies at their source, thereby intervening in the disease process.

However, despite its strong pathophysiological rationale, the actual role and efficacy of RTX in the clinical management of anti-GBM disease remain subjects requiring further investigation. The current challenges primarily stem from the following aspects. First, the relative rarity of the disease makes it difficult to conduct large-scale, prospective, randomized controlled trials. Most available evidence is limited to retrospective case series or individual reports, which generally represent a low level of evidence in evidence-based medicine. Second, conclusions from existing studies are inconsistent. Some studies have reported positive outcomes in terms of induction and maintenance of remission (6, 7), particularly when RTX is used as an alternative to traditional cyclophosphamide-based regimens in younger patients or those with fertility concerns (8). However, other observations indicate that its efficacy is not universally significant, especially in critically ill patients who have progressed to end-stage renal disease (ESRD) (9), where renal benefits appear limited. This uncertainty in therapeutic efficacy may be closely related to heterogeneity in patient populations and differences in treatment timing. Therefore, further clinical studies are urgently needed to validate the efficacy of RTX in anti-GBM disease.

This study aims to systematically review the clinical data of patients with anti-GBM disease treated at our center, comprehensively analyze the relationship between clinicopathological characteristics and prognosis, and further evaluate the real-world effectiveness of RTX in the treatment of this condition. Adopting a retrospective cohort design, the study focuses on comparing patients subjected to RTX-based treatment with those receiving conventional immunosuppressive regimens, with emphasis on differences in induction of remission, renal survival, and overall outcomes. The findings are expected to provide evidence-based support for optimizing individualized treatment strategies for this rare disease.

## 2 Materials and methods

### 2.1 Study population

**Figure 1:**
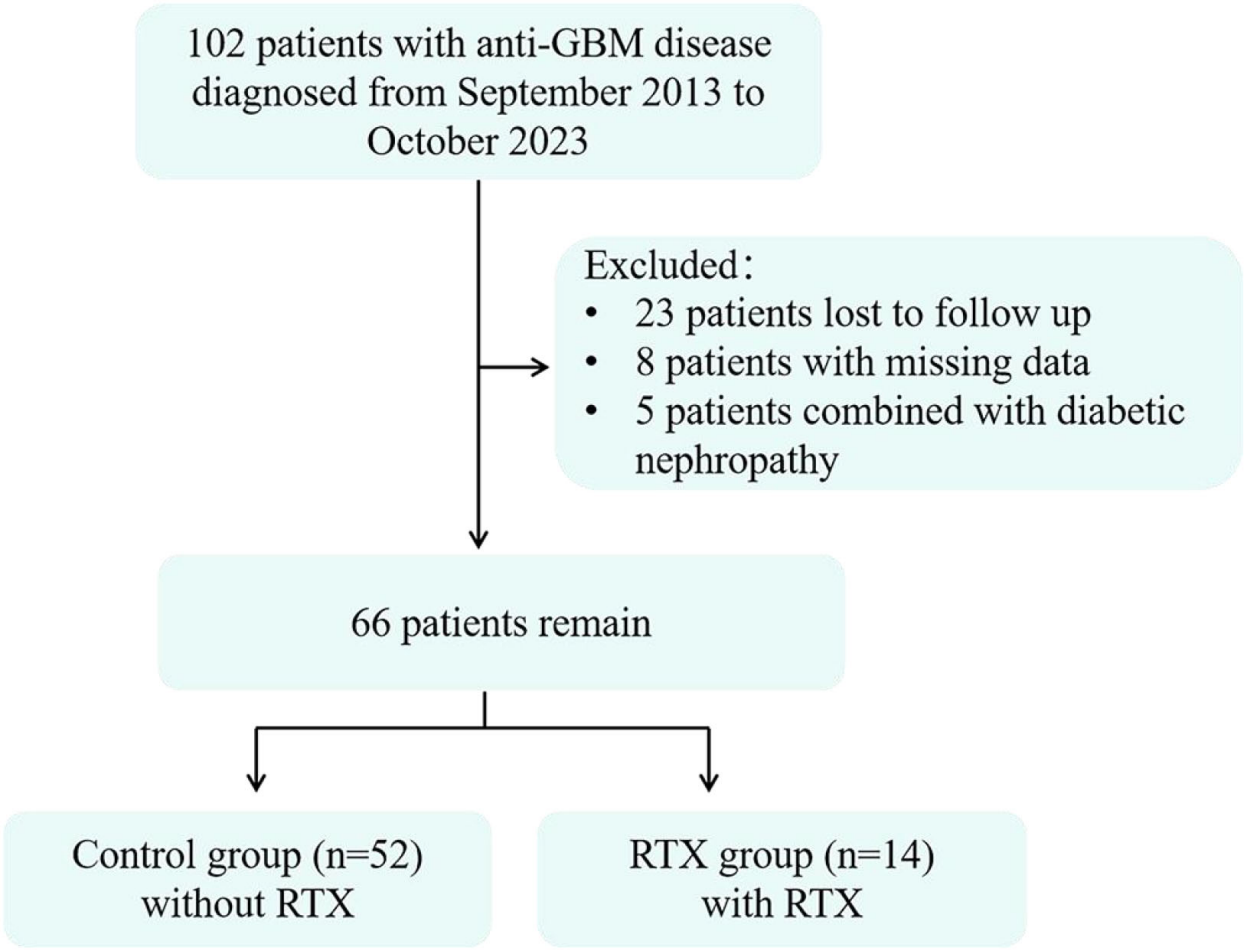
Patient enrollment flowchart.

This study was conducted in the Department of Nephrology at the First Affiliated Hospital of Zhengzhou University. A total of 102 patients diagnosed with anti-GBM disease were initially screened between September 2013 and October 2023. After the exclusion of 36 patients based on predefined criteria, 66 eligible adult patients (age ≥18 years) were ultimately included in the final analysis. The detailed patient recruitment process is illustrated in **Figure 1**. The study was approved by the Ethics Committee of Zhengzhou University First Affiliated Hospital (2025-KY-2076-001) and abided by the Declaration of Helsinki. Patient informed consent was waived, as this was a retrospective study.

### 2.2 Treatment protocol

Patients in the control group received conventional therapy, including plasma exchange, glucocorticoids, and cyclophosphamide, administered according to clinical indications and patient preference. Patients in the RTX group received the same conventional therapy as the control group, with the addition of RTX. RTX was administered intravenously according to one of two regimens: 1g on days 1 and 15 (n=5), or 375 mg/m² once weekly for 4 consecutive weeks (n=9). The choice of regimen was at the treating physician’s discretion, based on patient age, body surface area, and clinical judgment.PE Duration: PE was maintained until the following criteria were met: 1) serological clearance of anti-GBM antibodies (as evidenced by consecutive negative titers), 2) clinical evidence of resolving glomerular injury, defined by improvement or stabilization of serum creatinine, reduction of hematuria/proteinuria, or dialysis independence, 3) resolution of pulmonary hemorrhage, or 4) clinical justification for PE was no longer applicable(e.g., advanced irreversible chronic changes on biopsy with no pulmonary involvement).

### 2.3 Data collection

#### 2.3.1 Data source and variables

Study data were collected from the Electronic Health Record system, encompassing demographics, laboratory results, renal pathology reports, immunosuppressive regimens, renal replacement therapy, and PE.

#### 2.3.2 Laboratory parameters

Baseline laboratory values, obtained on admission day 1 or 2, included anti-GBM antibody titers, hemoglobin (Hb) concentration, serum creatinine level, B-type natriuretic peptide (BNP) level, albumin level, urinary protein-to-creatinine ratio (UPCR), C-reactive protein (CRP) level, erythrocyte sedimentation rate (ESR), and ANCA titers.

#### 2.3.3 Pathological assessment

Renal biopsy findings were evaluated for the percentage of glomeruli with crescents, the severity of tubulointerstitial damage, and any concurrent renal diseases.

Follow-up Data: Outcome data were collected via meticulous medical chart reviews and supplementary telephone follow-ups.

### 2.4 Statistical analysis

#### 2.4.1 Continuous data

Normally distributed variables are presented as mean ± standard deviation and were compared using the Student’s t-test. Non-normally distributed variables are reported as median (Q_1_, Q_3_) and were compared using the Mann-Whitney U test or Kruskal-Wallis test.

#### 2.4.2 Categorical data

Presented as counts and/or percentages and compared using Fisher’s exact test.

#### 2.4.3 Survival analysis

Renal survival was analyzed with Kaplan-Meier curves, and comparisons were made with the log-rank test.

All analyses were performed using R software version 4.3.0 and GraphPad Prism (v10.0). Statistical significance was defined as p < 0.05.

## 3 Results

### 3.1 Demographic and clinical data

A total of 66 patients with anti-GBM disease were included in this study. The cohort was stratified into two groups based on treatment: the control group (n=52) received standard therapy without RTX, and the RTX group (n=14) received standard therapy with RTX.

The baseline demographic, serological, and clinical characteristics of both groups are summarized in **Table 1**. No statistically significant differences were observed between the two groups at presentation. Specifically, the groups were comparable in terms of gender distribution, age, and key disease severity indicators, including anti-GBM antibody levels (494.23 ± 265.70 vs. 491.00 ± 238.31 IU/ml, p=0.969), initial serum creatinine (727.50 vs. 592.50 μmol/L, p=0.556), and the incidence of diffuse alveolar hemorrhage (7.69% vs. 14.29%, p=0.812). Furthermore, the proportion of patients who were ANCA-positive (17.31% vs. 21.43%, p=1.000) and the rate of dialysis-dependency at initial presentation (73.08% vs. 71.43%, p=1.000) were similar between the control and RTX groups.

**Table 1:** Clinical characteristics of all patients.

Variables	Total (n = 66)	Control (n = 52)	RTX (n = 14)	*P*
Gender [male, % (n)]	27 (40.91)	20 (38.46)	7 (50.00)	0.436
Age (years)	49.00 (33.50, 61.50)	48.00 (36.00, 60.50)	54.00 (32.75, 63.50)	0.790
Anti-GBM antibody levels, IU/ml	493.59 ± 258.63	494.23 ± 265.70	491.00 ± 238.31	0.969
Initial creatinine, μmol/L	704.50 (383.25, 1021.25)	727.50 (466.50, 1004.00)	592.50 (344.00, 973.75)	0.556
Diffuse alveolar hemorrhage, n(%)	6 (9.09)	4 (7.69)	2 (14.29)	0.812
Positive ANCA, n (%)	12 (18.18)	9 (17.31)	3 (21.43)	1.000
MPO, n (%)	12 (100)	9 (100)	3 (100)	1.000
MPO levels, RU/ml	471.50 (223.50, 944.75)	477.00 (463.00, 1055.00)	216.00 (192.75, 562.00)	0.373
PR3, n(%)	0 (0)	0 (0)	0 (0)	
Hb, g/L	83.00 (75.00, 95.75)	80.50 (74.50, 94.25)	90.00 (81.50, 101.00)	0.167
BNP, pg/ml	7365.00 (2678.00, 13764.00)	8238.00 (3027.25, 13472.50)	3653.00 (1842.00, 11971.00)	0.230
UPCR, g/g	3.60 (2.15, 6.39)	3.95 (2.38, 6.71)	2.71 (1.56, 4.15)	0.253
Serum albumin, g/L	29.07 ± 4.02	28.73 ± 4.03	28.73 ± 4.03	0.194
CRP, mg/L	56.44 (23.55, 96.50)	55.42 (23.11, 86.90)	76.33 (23.67, 104.00)	0.616
ESR, mm/h	107.00 (82.00, 133.00)	102.00 (72.75, 130.75)	108.00 (87.00, 139.00)	0.759
Time between onset and diagnosis, days	15.00 (7.00, 24.00)	15.00 (7.00, 25.50)	12.00 (7.75, 20.00)	0.581
Dialysis-dependency at initial presentation, n(%)	48 (72.73)	38 (73.08)	10 (71.43)	1.000
Glucocorticoids, n(%)	66 (100)	52(100)	14(100)	1.000
Glucocorticoids Pulse,n(%)	44 (66.67)	37 (71.15)	7 (50.00)	0.242
Cyclophosphamide, n(%)	40 (60.61)	33 (63.46)	7 (50.00)	0.360
Cumulative dose of Cyclophosphamide (mg)	4250(3200, 5775)	4600 (3200, 6000)	3600 (2800, 5400)	0.485
Plasma exchange, n(%)	63 (95.45)	50 (96.15)	13 (92.86)	0.517
Number of plasma exchange sessions	10.00 (6.00, 13.00)	10.00 (6.00, 12.00)	12.00 (9.00, 13.00)	0.215

Anti-GBM, anti-glomerular basement membrane; ANCA, Anti-neutrophil cytoplasmic antibody; MPO, myeloperoxidase; PR3, Proteinase 3; Hb, hemoglobin; BNP, B-type natriuretic peptide; UPCR, urinary protein-to-creatinine ratio; CRP, C-reactive protein; ESR, erythrocyte sedimentation rate.

Concomitant immunosuppressive therapies were also balanced across the groups. All patients received glucocorticoids, and the utilization rates of pulse glucocorticoids (71.15% vs. 50.00%, p=0.242), cyclophosphamide (63.46% vs. 50.00%, p=0.360), and plasma exchange (96.15% vs. 92.86%, p=0.517) showed no significant differences. These findings confirm that the two groups were well-matched in their baseline characteristics and initial management, facilitating a more valid comparison of subsequent outcomes.

### 3.2 Pathological characteristics

Of the 66 patients included in the study, 31 individuals (22 in the control group and 9 in the RTX group) underwent renal biopsy for pathological evaluation. The baseline pathological characteristics of these patients are summarized in **Table 2**.

**Table 2:** Pathological characteristics of patients with renal biopsy.

Variables	Total (n = 31)	Control (n = 22)	RTX (n = 9)	*P*
Crescent formation, n (%)	31(100)	22(100)	9(100)	1.000
Percentage of crescents (%)	70.96 ± 20.43	74.30 ± 16.48	62.81 ± 27.33	0.1587
Percentage of Cellular crescents (%)	41.92 ± 27.15	44.53 ± 25.40	35.56 ± 31.73	0.413
Percentage of Cellular-fibrous crescents (%)	11.40 (0.30, 17.90)	11.85 (1.25, 17.82)	11.10 (0.00, 17.60)	0.775
Percentage of Fibrous crescents (%)	0.25 (0.00, 22.25)	0.82 (0.00, 22.78)	0.00 (0.00, 19.10)	0.764
Percentage of Necrosis(%)	0.00 (0.00, 13.20)	0.00 (0.00, 7.10)	0.00 (0.00, 18.20)	0.698
Severe renal interstitial pathology, n(%)	21 (67.74)	16 (72.73)	5 (55.56)	0.251
With IgAN, n(%)	10 (32.26)	8 (36.36)	2 (22.22)	0.677
With MN, n(%)	13 (41.94)	8 (36.36)	5 (55.56)	0.433

IgAN, IgA nephropathy; MN, membranous nephropathy.

Crescent formation, the hallmark pathological lesion of anti-GBM disease, was observed in all biopsied patients (100%). Further analysis of crescent composition revealed no significant differences between the two groups. The proportion of cellular crescents (RTX group vs. control group: 35.56% ± 31.73% vs. 44.53% ± 25.40%; p=0.413), fibrocellular crescents [median: 11.10% vs. 11.85%; p=0.775], and fibrous crescents [median: 0.00% vs. 0.82%; p=0.764] were all comparable. Furthermore, no statistically significant differences were found in the proportion of glomerular necrosis or the incidence of severe renal interstitial pathology (55.56% vs. 72.73%; p=0.251).

Regarding coexisting glomerular diseases, the prevalence of comorbid IgA nephropathy (IgAN) (22.22% vs. 36.36%; p=0.677) and membranous nephropathy (MN) (55.56% vs. 36.36%; p=0.433) was similar between the RTX and control groups, with no statistical significance.

These pathological data demonstrate that the patients in the RTX and control groups were well-balanced in terms of the severity of renal pathological injury, crescent type, and spectrum of comorbid diseases at baseline. This comparability eliminates potential bias in efficacy assessment due to uneven initial pathological damage.

### 3.3 Clinical outcomes

As detailed in **Table 3**, the analysis of patient outcomes revealed potential benefits of RTX therapy. Specifically, although the RTX group demonstrated only numerically favorable trends in the rate of dialysis discontinuation (50.0% vs. 21.05%, p=0.107) and the 3-year ESRD incidence (42.86% vs. 63.46%, p=0.223) without reaching statistical significance, a clear positive signal was observed. A key finding from **Table 3** is that the median time to dialysis discontinuation was significantly shorter in the RTX group compared to the control group (26 days vs. 41 days, p=0.009), suggesting that RTX treatment may significantly accelerate the recovery of renal function. However, among the 13 patients who achieved dialysis independence, those who progressed to ESRD within 3 years (n=3) had a median time to dialysis independence of 42 days, compared with 35 days in those who remained ESRD-free (n=10), with no significant difference (p=0.189) (**Supplementary Table 1**). Thus, faster dialysis independence did not predict lower 3-year ESRD risk in this cohort.

**Table 3:** Outcomes of all patients.

Variables	Total (n = 66)	Control (n = 52)	RTX (n = 14)	*P*
Dialysis-dependency at initial presentation, n(%)	48 (72.73)	38 (73.08)	10 (71.43)	1.000
Percentage of discontinuing dialysis, n(%)	12 (25.00)	8 (21.05)	5 (50)	0.107
Time to dialysis discontinuation, days	36.00 (28.00, 43.00)	41.00 (35.25, 51.00)	26.00 (23.00, 35.00)	0.009*
ESRD at 3 years, n(%)	39(59.09)	33(63.46)	6(42.86)	0.223
Infection, n(%)	22 (33.33)	18 (34.62)	4 (28.57)	0.915
Death,n(%)	6 (9.09)	5 (9.62)	1 (7.14)	1.000

ESRD, end-stage renal disease. *P<0.05.

Significant *P-values (<0.05)* are represented in bold.

**Figure 2:**
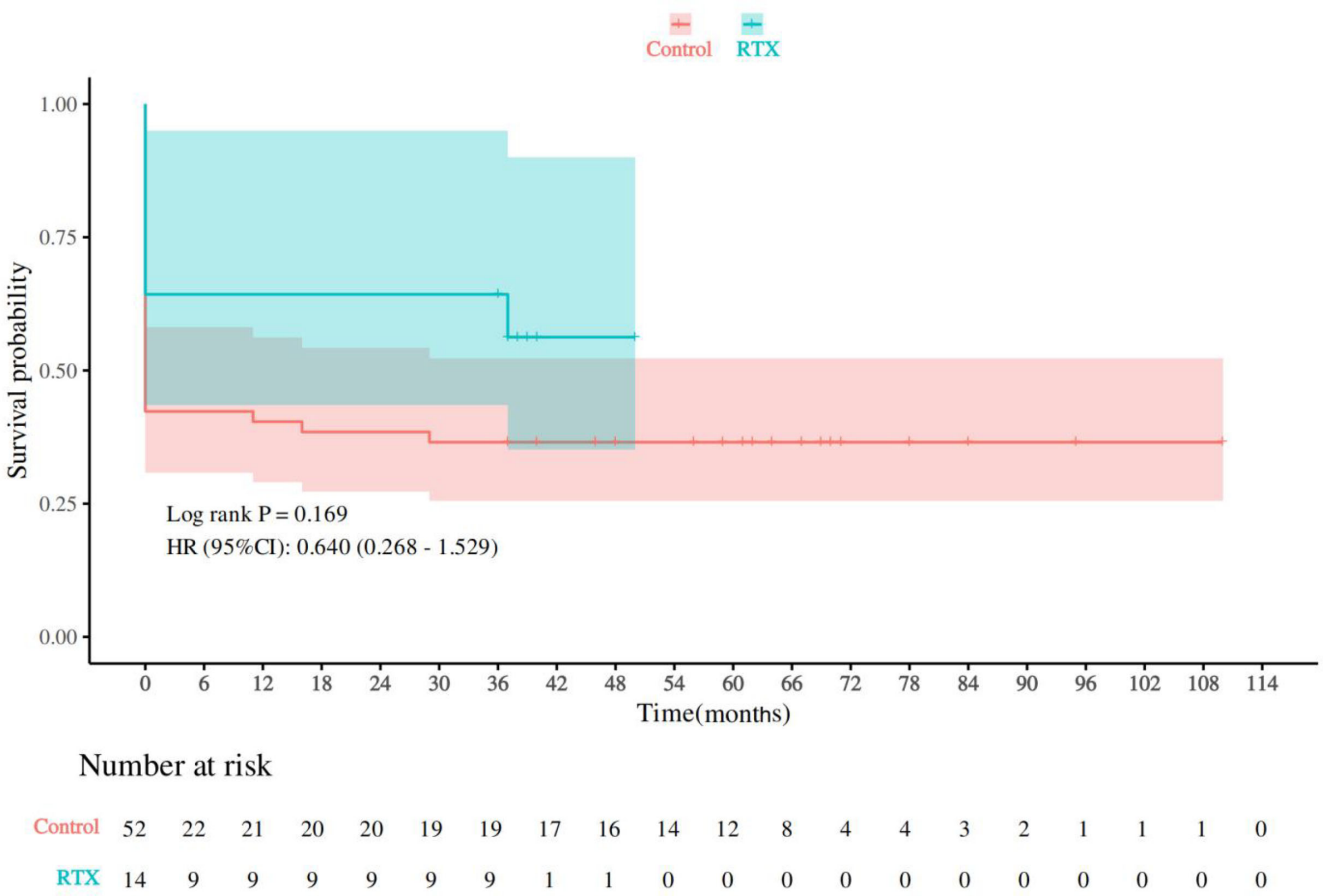
Kaplan-Meier analysis of renal survival in anti-GBM disease patients stratified by RTX treatment. The Kaplan-Meier curves demonstrated a numerically higher renal survival rate at 3 years in the RTX-treated group compared to the control group, with an absolute difference of approximately 36%. However, the difference between the two groups was not statistically significant (Log-rank p=0.169; HR = 0.640, 95% CI: 0.268–1.529).

The Kaplan-Meier curve also demonstrated a trend toward higher survival probability in the RTX group compared to the control group (**Figure 2**); however, this difference did not reach statistical significance by the Log-rank test (p=0.169). The hazard ratio (HR) was 0.640 (95% CI [0.268, 1.529]), suggesting a potential 36% reduction in the risk of death with RTX treatment, although this effect remains uncertain and requires further validation in larger studies.

### 3.4 Prognostic factors for 3-year ESRD status

Stratification by ESRD status at 3 years revealed significant differences in multiple baseline characteristics between the groups. Data from **Table 4** showed that patients who progressed to ESRD (n=39), compared to those who remained ESRD-free (n=27), presented with significantly worse renal function at diagnosis (initial creatinine: 889.00 vs. 353.00 μmol/L, p<0.001), more severe anemia (Hb: 78.00 vs. 92.00 g/L, p=0.007), lower serum albumin (28.10 ± 4.14 vs. 30.44 ± 3.47 g/L, p=0.019), and markedly elevated BNP levels (11550.00 vs. 2577.00 pg/ml, p<0.001). Furthermore, the proportion of patients requiring dialysis at presentation was drastically higher in the ESRD group (97.44% vs. 37.04%, p<0.001), and they received cyclophosphamide treatment significantly less frequently (48.72% vs. 77.78%, p=0.018). To assess whether control patients who did not receive cyclophosphamide had worse outcomes, we performed a subgroup analysis within the control group (**Supplementary Table 2**). The results showed no significant difference in dialysis independence rate or time to dialysis independence between those who received cyclophosphamide and those who did not, indicating that the heterogeneity in cyclophosphamide use did not skew the main comparison. Male gender was also identified as a risk factor associated with progression to ESRD (51.28% vs. 25.93%, p=0.039).

**Table 4:** Clinicopathological characteristics of patients based on ESRD status at 3 years.

Variables	Total (n = 66)	ESRD-free at 3 years (n = 27)	ESRD at 3 years (n = 39)	*P*
Gender [male, % (n)]	27 (40.91)	7 (25.93)	20 (51.28)	0.039*
Age (years)	49.00 (33.50, 61.50)	55.00 (46.00, 59.00)	46.00 (30.00, 66.00)	0.309
Time between onset and diagnosis, days	15.00 (7.00, 24.00)	20.00 (10.00, 28.00)	11.00 (7.00, 20.00)	0.076
Anti-GBM antibody levels, IU/ml	493.59 ± 258.63	449.94 ± 264.99	526.03 ± 252.72	0.259
MPO, n(%)	12 (18.18)	5 (18.52)	7 (17.95)	1.000
Hb, g/L	83.00 (75.00, 95.75)	92.00 (81.50, 99.00)	78.00 (70.50, 89.00)	0.007*
Initial creatinine, μmol/L	704.50 (383.25, 1021.25)	353.00 (263.50, 701.50)	889.00 (694.50, 1197.50)	**<.001**
Serum albumin, g/L	29.07 ± 4.02	30.44 ± 3.47	28.10 ± 4.14	0.019*
UPCR, g/g	3.60 (2.15, 6.39)	2.77 (1.81, 4.83)	4.80 (2.65, 7.73)	0.072
CRP, mg/L	56.44 (23.55, 96.50)	54.40 (15.60, 76.33)	56.48 (33.05, 99.62)	0.238
ESR, mm/h	107.00 (82.00, 133.00)	114.00 (73.00, 139.00)	96.50 (83.50, 126.25)	0.591
BNP, pg/ml	7365.00 (2678.00, 13764.00)	2577.00 (1108.00, 4325.50)	11550.00 (6030.75, 21017.50)	<.001*
Percentage of Cellular crescents (%)	50.55 (20.82, 62.58)	50.00 (16.70, 62.50)	51.10 (27.10, 62.65)	0.953
Percentage of Cellular-fibrous crescents (%)	11.25 (0.00, 17.75)	11.40 (0.00, 18.20)	6.70 (0.30, 15.95)	0.547
Percentage of Fibrous crescents (%)	0.12 (0.00, 21.72)	0.25 (0.00, 19.10)	0.00 (0.00, 42.45)	0.641
Percentage of Necrosis(%)	0.00 (0.00, 15.28)	0.00 (0.00, 18.20)	0.00 (0.00, 5.55)	0.743
Severe renal interstitial pathology, n(%)	21/31	12/19	9/12	0.697
With IgAN, m/n	10/31	8/19	2/12	0.240
With MN, m/n	13/31	7/19	6/12	0.710
Number of plasma exchange sessions	10.00 (6.00, 13.00)	10.00 (6.25, 10.75)	11.50 (6.50, 14.00)	0.045*
Dialysis-dependency at initial presentation, n(%)	48 (72.73)	10 (37.04)	38 (97.44)	<.001*
Glucocorticoids Pulse,n(%)	44 (66.67)	21 (77.78)	23 (58.97)	0.111
Cyclophosphamide, n(%)	40 (60.61)	21 (77.78)	19 (48.72)	0.018*
RTX, n(%)	14 (21.21)	8 (29.63)	6 (15.38)	0.164
Days from diagnosis to RTX initiation, days	18.50 (12.00, 25.50)	18.50 (11.75, 23.00)	18.00 (12.00, 30.00)	0.604
Infection, n(%)	22 (33.33)	6 (22.22)	16 (41.03)	0.111

Anti-GBM, anti-glomerular basement membrane; MPO, myeloperoxidase; Hb, hemoglobin; BNP, B-type natriuretic peptide; CRP, C-reactive protein; ESR, erythrocyte sedimentation rate; IgAN, IgA nephropathy; MN, membranous nephropathy; RTX, rituximab. *P<0.05.

Significant *P-values (<0.05)* are represented in bold.

## 4 Discussion

This study retrospectively analyzed the clinical data of 66 patients with anti-GBM disease, focusing on evaluating the efficacy of RTX combined with standard therapy and exploring prognostic factors affecting the 3-year renal survival rate. The results showed that although the RTX group exhibited only numerical advantages in dialysis independence rate (50.0% vs. 18.4%, p=0.094) and 3-year ESRD incidence (42.86% vs. 63.46%, p=0.223), the median time to dialysis independence was significantly shorter in the RTX group (26 days vs. 41 days, p=0.009), suggesting that RTX may accelerate renal function recovery. Furthermore, initial dialysis dependence was identified as the strongest risk factor for progression to ESRD (97.44% vs. 37.04%, p<0.001). Interestingly, although RTX significantly shortened the time to dialysis independence, this accelerated recovery was not associated with a reduced risk of ESRD at 3 years. Larger studies with longer follow-up are needed to further explore this relationship.

The significantly shorter median time to dialysis independence observed in the RTX group may be related to the rapid B cell depletion and subsequent inhibition of pathogenic antibody production induced by RTX. In a study involving 39 anti-GBM disease patients, Chen et al. found that the rate of anti-GBM antibody decline at 0.5 months post-treatment was significantly higher in the group receiving RTX combined with standard therapy compared to the control group (75.3% ± 17.6% vs. 56.3% ± 18.3%, p=0.018) (7). That study further demonstrated that patients who did not progress to ESRD had a faster rate of antibody decline, suggesting that early and rapid clearance of pathogenic antibodies is crucial for improving renal outcomes. Although a direct comparison of antibody decline rates was not performed in the present study, the significantly shortened time to dialysis independence indirectly supports this mechanism.

Furthermore, RTX may exert immunomodulatory effects by influencing T-cell function. B-cell depletion can alter T-cell numbers, activation status, and regulatory T-cell ratios, thereby mitigating local inflammatory responses (10). In anti-GBM disease, a rapidly progressive condition characterized by crescent formation, early intervention in the inflammatory cascade is crucial for preserving residual renal function. In this study, patients in the RTX group received treatment at an early stage of the disease course, which may be associated with accelerated renal function recovery.

Although the RTX group showed a trend toward a lower 3-year ESRD incidence (42.86% vs. 63.46%), the difference did not reach statistical significance. This finding is consistent with several recent studies. In a systematic review of 67 anti-GBM disease patients treated with RTX, Ivkovic et al. reported a renal survival rate of 67%, although that study also lacked a direct control group for comparison (9). Van Daalen et al., in a long-term follow-up study of 123 patients with anti-GBM disease, reported a 5-year renal survival rate of only 34%, and identified initial dialysis dependence as the strongest prognostic factor (11). In the present study, 71.4% of patients in the RTX group were dialysis-dependent at baseline. This high-risk characteristic may have diluted the therapeutic benefit of RTX.

Notably, despite the severe initial condition (71.4% dialysis-dependent) in the RTX group, 50.0% had not progressed to ESRD by the end of follow-up, which was superior to the control group (9.1% non-progressors). This result aligns with the findings of Jaryal and Vikrant, in which among 3 patients receiving RTX as first-line therapy, one recovered from dialysis dependence to normal renal function, and another progressed only to CKD stage 3 (6). Similarly, in a cohort study of 107 patients, Liu et al. found that early initiation of plasma exchange (at lower serum creatinine level or lower anti-GBM antibody levels) was associated with better outcomes, highlighting the importance of treatment timing (12).In this study, baseline therapies (plasma exchange, cyclophosphamide, glucocorticoids) were comparable between the RTX and control groups. However, the shorter time to dialysis independence in the RTX group suggests that RTX as an “add-on” therapy may be particularly valuable for specific patient subgroups. Nevertheless, due to the small sample size (only 14 patients in the RTX group), this study may have been underpowered to detect statistically significant differences in long-term renal survival. Future validation through larger, multicenter studies is warranted.

Multivariate analysis in this study confirmed that initial dialysis dependence was the strongest risk factor for progression to ESRD (97.44% vs. 37.04%, p<0.001), a result highly consistent with previous literature. The study by van Daalen et al. showed a 5-year renal survival rate of only 8% for patients dialysis-dependent at presentation, compared to 82% for those not dialysis-dependent (11). As early as 2001, Levy et al. reported that no patient with initial dialysis dependence and 100% cellular crescents on renal biopsy recovered renal function (13).

This study further revealed that patients progressing to ESRD not only had higher initial creatinine levels, more severe anemia, and more pronounced hypoalbuminemia but also exhibited significantly elevated BNP levels. This suggests that cardiorenal syndrome or volume overload may contribute to poor prognosis. Notably, the proportion of patients receiving cyclophosphamide treatment was significantly lower in the ESRD group compared to the non-ESRD group (48.72% vs. 77.78%, p=0.018). This may reflect clinicians’ concerns regarding the use of immunosuppressants in patients with severe renal failure. However, the study by Jaryal and Vikrant demonstrated that even patients initially dependent on dialysis could achieve renal recovery with aggressive use of RTX combined with plasma exchange (6). Therefore, initial dialysis dependence should not be an absolute criterion for withholding intensive immunosuppressive therapy but should be considered alongside other clinical and pathological indicators for comprehensive assessment.

No serious adverse events were reported in the RTX group in this study, consistent with the favorable safety profile of RTX documented in prior literature. Compared to cyclophosphamide, RTX avoids risks such as gonadal toxicity, hemorrhagic cystitis, and long-term malignancy (14), making it particularly suitable for young patients and those requiring fertility preservation.

## 5 Limitations

This study has several limitations. Its single-center, retrospective design may introduce selection bias. The limited sample size in the RTX treatment group might obscure its true clinical efficacy. Treatment decisions (e.g., RTX administration) were non-randomized and could have been influenced by disease severity and physician preference. Although baseline comparisons indicated group balance, the possibility of unmeasured confounding factors cannot be excluded.

## 6 Conclusions

In summary, the findings of this study indicate that the addition of RTX to standard therapy may provide benefits in terms of faster renal function recovery in patients with anti-GBM disease, particularly by potentially accelerating the process of dialysis discontinuation. Renal insufficiency at the time of diagnosis is the most critical predictor of long-term renal survival. Larger prospective studies are required in the future to clarify the definitive role of RTX within the therapeutic spectrum of this disease.

## Data Availability

The raw data supporting the conclusions of this article will be made available by the authors, without undue reservation.

## Supplementary material

Supplementary Table 1 Association between time to dialysis independence and 3-year ESRD status among patients who achieved dialysis independence (n=13). ESRD, end-stage renal disease.

Supplementary Table 2 Dialysis independence outcomes in the control group stratified by cyclophosphamide use. Among control patients with available follow-up data, no statistically significant difference was observed in the rate of dialysis independence between those who received cyclophosphamide and those who did not (p=0.258, Fisher’s exact test). The median time to dialysis independence was also similar (43 days vs. 42 days).
