# Supplementary material for: Rituximab is associated with accelerated dialysis independence in anti-glomerular basement membrane disease: a retrospective cohort analysis of renal survival
Source: Front Immunol. 2026 May 11;17:1835889. doi: 10.3389/fimmu.2026.1835889 (PMC13199269; doi:10.3389/fimmu.2026.1835889)
Supplement: Supplementary Table 1 — Association between time to dialysis independence and 3-year ESRD status among patients who achieved dialysis independence (n=13). ESRD, end-stage renal disease. [file DataSheet1.docx]

Table.S1 Association between time to dialysis independence and 3-year ESRD status among patients who achieved dialysis independence (n=13)

|  | ESRD-free at 3 years (n = 10) | ESRD at 3 years  (n = 3) | *P* |
| --- | --- | --- | --- |
| Median time to dialysis independence, days (IQR) | 42 (36, 56) | 35 ( 25.5, 43.5) | 0.189 |

ESRD, end-stage renal disease.

Table.S2 Dialysis independence outcomes in the control group stratified by cyclophosphamide use

|  | CTX (n =33) | Without CTX (n = 21) | *P* |
| --- | --- | --- | --- |
| Dialysis-dependency at initial presentation, n(%) | 21 (63.64) | 17 (80.95) | 0.229 |
| Percentage of discontinuing dialysis, m/n | 6/21 | 2/17 | 0.258 |
| Median time to dialysis independence, days (range) | 40 (35–52) | 42 (36–48) | - |

Among control patients with available follow-up data, no statistically significant difference was observed in the rate of dialysis independence between those who received cyclophosphamide and those who did not (p=0.258, Fisher’s exact test). The median time to dialysis independence was also similar (43 days vs. 42 days).
